# Hydrogen and Deuterium Molecular Escape from Clathrate Hydrates: “Leaky” Microsecond-Molecular-Dynamics Predictions

**DOI:** 10.1021/acs.jpcc.1c00987

**Published:** 2021-04-09

**Authors:** Yogeshwaran Krishnan, Mohammad Reza Ghaani, Niall J. English

**Affiliations:** School of Chemical and Bioprocess Engineering, University College Dublin, Belfield, Dublin 4, Ireland

## Abstract

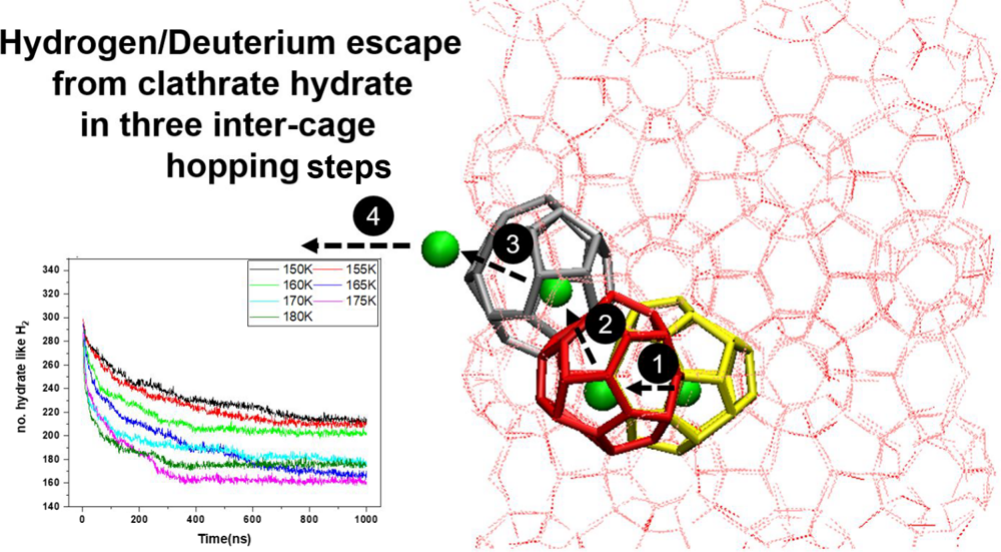

It is predicted herewith that the leakage of both hydrogen (H_2_) and deuterium (D_2_) from sII clathrate hydrates, borne of guest chemical-potential equalization driving enhanced nonequilibrium intercage hopping, should be observable experimentally. To this end, we have designed simulations to realize and study this process by microsecond molecular dynamics within the temperature range of 150–180 K—for which the hydrate lattice was found to be stable. In this pursuit, we considered initial large-cage (5^12^6^4^) guest occupancies of 1–4, with single occupation of 5^12^ cavities. Examining transient, nonequilibrium intercage hopping, we present a lattice-escape activation energy for the four nominal large-cage occupancies (1–4), by fitting to the hydrate-leakage rate. The intercage hopping of H_2_ and D_2_ was studied using Markov-chain models and expressed at different temperatures and large-cage occupancies. The free energy of guest “binding” in the large and small cages was also computed for all of the occupancies. Toward equilibrium, following the majority of H_2_/D_2_ escape via leakage, the percentage of occupancies was calculated for both H_2_ and D_2_ for all of the systems for all initial nominal large-cage occupancies; here, not unexpectedly, double occupancies occurred more favorably in large cages and single occupancies dominated in small cages.

## Introduction

Natural gas hydrates are compounds of nonstoichiometric inclusion in which water molecules form cages, or cavities, which entrap gas molecules—known as guests. The hydrogen bonding of water molecules^1^ binds these cages together in crystal lattices, not dissimilar to kinds of “cagey” ice, the configurations of which depend on the sizes of the guest molecules. The three most commonly occurring types, or polymorph, of crystal structure have been labeled as sI, sII, and H. Each polymorph has various crystallographic characteristics, including cavities of various shapes and sizes. In the sI (type I) case, a clathrate contains 46 water molecules in a unit cell that form two pentagonal dodecahedra (5^12^ cages) and six truncated hexagonal trapezoidal cavities (5^12^6^2^). In the case of type II (sII), 136 water molecules are composed of sixteen 5^12^ and eight 5^12^6^4^ cages in a unit cell. The type H clathrate is made up of 36 water molecules in a unit cell containing three 5^12^, two 4^3^5^6^6^3^, and one 5^12^6^8^ cages. In which the host are water molecules and the guest molecules are gases, these clathrate hydrates act as host complexes. The structure of a clathrate hydrate can collapse into liquid water all too easily without the help of the trapped gas molecules.^2^

Methane hydrate is the most common of these crystalline compounds, and the world’s natural gas-hydrate (NGH) reserves represent a large supply of hydrocarbons. However, given that gas hydrates have various potential applications, many other industrial forms of clathrate center around water desalination, carbon-dioxide sequestration, and the preservation of certain molecules, such as hydrogen.^3^ Importantly, during both hydrate formation and decomposition, clathrate hydrates do not produce any chemical waste, and hydrogen-natural gas mixtures can also be used as an energy resource in and of themselves.^4^ Indeed, due to their potential as economic and environmentally friendly hydrogen storage materials, hydrogen clathrate hydrates have attracted a great deal of interest in recent years.^5^

Dyadin et al. were the first to describe simple hydrogen clathrate hydrates, which only have hydrogen molecules as guests,^6^ which were later characterized in more detail by Mao et al.^7^ It was reported by Mao et al. that the small cage is typically occupied by two H_2_ molecules and four by the large hydrogen hydrate cage at moderate to high pressures, equivalent to 5.3 wt % molecular hydrogen in the material.^7^ A subsequent neutron-diffraction analysis of pure sII hydrogen hydrate by Lokshin et al.^8^ confirmed the quadruple D_2_ occupancy of the broad cage at moderate to high pressures below 70 K but found only one D_2_ molecule in the small cage, reducing the estimation of the maximum storage capacity of hydrogen to about 3.8 wt %. The same study showed that at ambient pressure, the large cages began to lose D_2_ molecules above 70 K and above 190 K at 200 MPa; the lowest occupancy of two D_2_ molecules was calculated for the large cage. Also, they have shown in their experiments that the small cage can hold only one H_2_ molecule, while the wide cage can encapsulate up to four H_2_ molecules.^8^ In our previous study,^9^ we performed molecular-dynamics (MD) calculations on both D_2_ and H_2_ in bulk hydrate for cage occupancies and their self-diffusivity via intercage hopping and studied using a Markov-chain model these hopping phenomena as a function of temperature. In addition, turning to intramolecular motion, Sebastianelli et al. have studied the quantum translational-rotational dynamics H_2_ and D_2_ clusters in sII large cages (5^12^6^4^),^10^ and Xu et al. reported the quantum translation-rotation studies of the small cage of sII type clathrate hydrate.^11^ Indeed, guest molecules in clathrate hydrates have been studied extensively using both theoretical^2,12−24^ and experimental^6,8,33,25−33^ techniques, and still there is a much scope to understand the properties of gas molecules in the interface of clathrate hydrates. In particular, given our recent study on “leaky” sII hydrates (of neon) via MD, allowing for hydrate-emptying by transfer to a neighboring vacuum,^34^ there are many open questions on guest molecules’ escape by intercage hopping—the underpinning mechanics of nonequilibrium molecular mass transfer and associated cage-level diffusivity and occupancy phenomena.

In the present study, given this broad and well-justified interest in hydrogen hydrates as a hydrogen-storage material, we are motivated by unresolved, open questions relating to both H_2_ and D_2_ intercage diffusivity and release of the H_2_ and D_2_ to vacuum, given our observation of “leaky” neon sII hydrates.^34^ We wish to understand if such leaks extend to H_2_ and D_2_. In particular, with a guest-loaded hydrate and therefore a chemical-potential driving force for guest mass transfer (via Fick’s Law), it is fascinating to scrutinize the phenomena underpinning transfer of guest molecules passing from one cage to another as a dramatic case study of nonequilibrium diffusion. Indeed, in the present study, we examine the cage-to-cage molecular hopping motions of H_2_ and D_2_ molecules inside a hydrate to a neighboring vacuum—both as a function of temperature and nominal guest occupation; in this, we determine the dynamical characteristics and interplay of cage occupancies and their associated activation-energy profiles in a Markov-chain framework,^9^ to gain better insights into the various aspects influencing escape of the guest molecules from their molecular “jail cells” to vacuum. In addition, to characterize driving force, the free-energy profile was also calculated using thermodynamic integration for all occupancies.^34^

## Methodology

**Figure 1:**
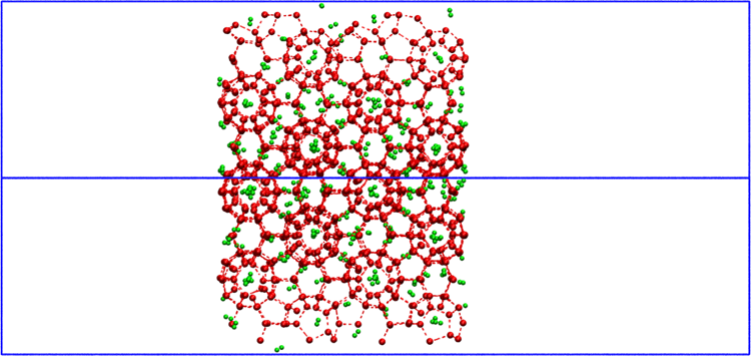
Schematic of a singly occupied (5^12^6^4^-cage) (110) plane H_2_/D_2_-bearing hydrate in the simulation box.

H_2_ and D_2_ molecules were put in 2 × 2 × 2 sII-clathrate supercells of vanishingly small dipole, with a size of 3.42 nm on each side, and the vacuum (with a length of 2 × 3.42 nm along the direction of heterogeneity) was applied on the x direction as shown in Figure 1; for *x*-, *y*-, and *z*-directions, periodic boundary conditions were applied, as in ref (34). H_2_ and D_2_ were put in 5^12^6^4^ large cages (i.e., featuring 12 pentagonal faces and 4 hexagonal ones) and small-cage 5^12^ dodecahedra. For each large cage, there were one to four (1, 2, 3, and 4) molecules placed therein, and a small cage held only one molecule as a guest (given the single small-cage occupation found by Lokshin et al.^8^). In this way, subsequent MD simulations were performed of either H_2_ or D_2_ as a guest with four different nominal initial concentrations for large-cage loading in the hydrate, so that the calculations were performed in four distinct configurations.

For both H_2_ and D_2_, the same force-field parameters were used in the previous MD investigation of sII hydrogen hydrates; the potentials are kept the same for both H_2_ and D_2_, and the extra masses are added to the D_2_ molecules; these replicated correctly the experimental gas-phase quadrupole moment of H_2_ and D_2_.^9^ MD simulations were carried out in the NVT ensemble using the 3D particle-mesh Ewald method to handle long-range interactions,^35^ with a Nosé–Hoover relaxation time of 5 ps. Simulations were conducted for 1 μs, using the velocity Verlet algorithm; Lennard-Jones 12-6 and real-space Ewald interactions were subject to a 1.2 nm cut-off. The GROMACS 5.1^36−38^ package was used for all simulations. Simulations were carried out at seven different temperatures, ensuring very carefully that the hydrate lattice itself remained intact throughout the full 1 μs, albeit subject to some surface-layer rearrangements at 175–180 K: 150, 155, 160, 165, 170, 175, and 180 K. Aside from visualization per se, we judged the number of enclathrated water and guest molecules using the Báez-Clancy (BC) geometric-recognition method, which distinguishes between water molecules in the hydrate, ice lattices, and liquid phase. This provided a quantitative check on lattice integrity, as well as allowing for a quantitative study of guest numbers’ depletion in the hydrate phase (in tandem with vacuum-phase H_2_/D_2_-molecule counting and visualization). Above around 180 K (i.e., in the 185–195 K region), there was the onset of bulk-lattice dissociation for the variously occupied hydrate-lattice systems (i.e., thermodynamic melting), albeit even after a good fraction of a microsecond, so simulations with these moderate thermodynamic thermal driving forces were not analyzed: as with ref (34)., the purpose of the present study is to investigate nonequilibrium cage-hop-mediated diffusional escape of H_2_/D_2_ guests under conditions in which the “leaky” lattice itself is thermodynamically (meta)stable and certainly kinetically so, over simulation timescales of at least a microsecond. As with ref (34). for the case of neon’s leaky escape from sII clathrate, this allows for plateaux in guest-release numbers to be realized (i.e., essentially attainment of guest chemical-potential equilibrium in both hydrate and vacuum phases).

In our calculations, the sII-clathrate lattice^39^ was used, and it is shown in Figure 1. The movement inside the clathrate-hydrate structure of H_2_ and D_2_ molecules was analyzed and captured as the Markov-chain model,^9,40^ and that facilitated by intercage hopping migration^41−43^ was analyzed and captured as Markov-chain models.^9,44^ It is necessary to remember that not all states can make a substantial contribution to the overall system’s configurational properties. Indeed, in producing a Markov chain,^44^ in principle, it is important to sample those states that make the most important contributions to accurately evaluate the system’s properties in the finite time available for the simulation, even though the 1 μs durations in the present study do allow for very extensive statistical sampling of cage-hop events in practice.

The Helmholtz (Δ*A*)/Gibbs free energy (Δ*G*) of H_2_/D_2_ “binding” in sII cavities (both large and small) were calculated with a leap-frog stochastic-dynamics integrator: the difference in free energy between two states of the system was determined using the coupling-parameter approach in conjunction with the thermodynamic-integration (TI) formulism. In this approach, the Hamiltonian *H* is modified as a function also of a coupling parameter, λ, i.e., *H* = *H* (p; q; λ), in such a way that λ = 0 describes system A (decoupled) and λ = 1 describes system B (coupled).^34^
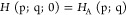

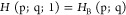
1

Further details are explained in ref (34). The intermediate λ values used in between decoupled and coupled states were varied from 0 to 1, in 11 steps. The calculations were run for up to 5 ns, sampled, and averaged during the overall 1 μs simulations. For statistical robustness, free-energy calculations were run for 10 different H_2_/D_2_ sites for both large (5^12^6^4^) and small (5^12^) cages.

## Results and Discussion

**Figure 2:**
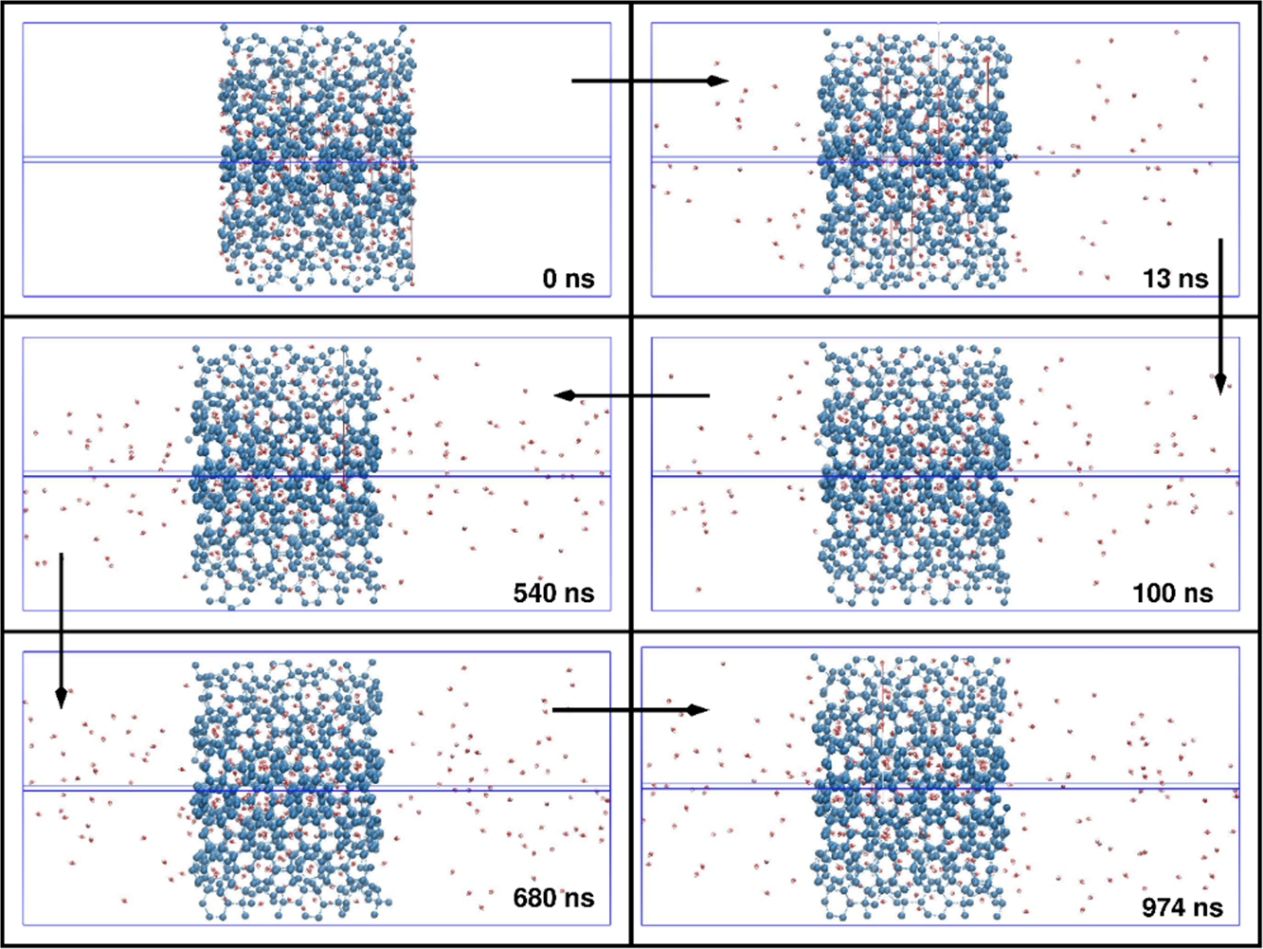
Snapshots of H_2_ release in from (doubly occupied 5^12^6^4^-cage) clathrate hydrates. Red denotes hydrogen, with release into the vacuum. Blue represents BC-classified hydratelike water molecules in the hydrate phase.

Given the fact that the present study considers D_2_ and H_2_ leakage below the melting point, that is, with the lattice itself fully intact, we used the BC approach to define the number of hydratelike water and D_2_ or H_2_ molecules—always being sure of avoiding lattice melting by 1 μs constancy of the enclathrated-water count, defined by the BC method. The H_2_ and D_2_ gas was arranged in four different configurations with a single occupancy in all small cages (5^12^) and one to four occupancies in a large cage in the system shown in Figure 1. As a representative example, shown in Figure 2 are some snapshots of the emptying of the (doubly occupied 5^12^6^4^-cage) H_2_-bearing hydrate; comparable ones for a similar D_2_ release are depicted in Figure S1 (see the Supporting Information). To remind, the overall system’s temperature was kept constant over 1 μs simulations using a Nosé–Hoover thermostat; two temperature trajectories are shown for the 5^12^6^4^-double-occupancy case in Figure S2 (cf. Supporting Information). A pair distance analysis performed between the guest molecules during one sampled 100 ns simulation period proved the free movement of the guest molecules inside the case and the absence of any dominant orientation between them. At each frame, depending on their distance, the molecules impose repulsive or attractive forces to the neighboring guest molecule (Figure S6).

**Figure 3:**
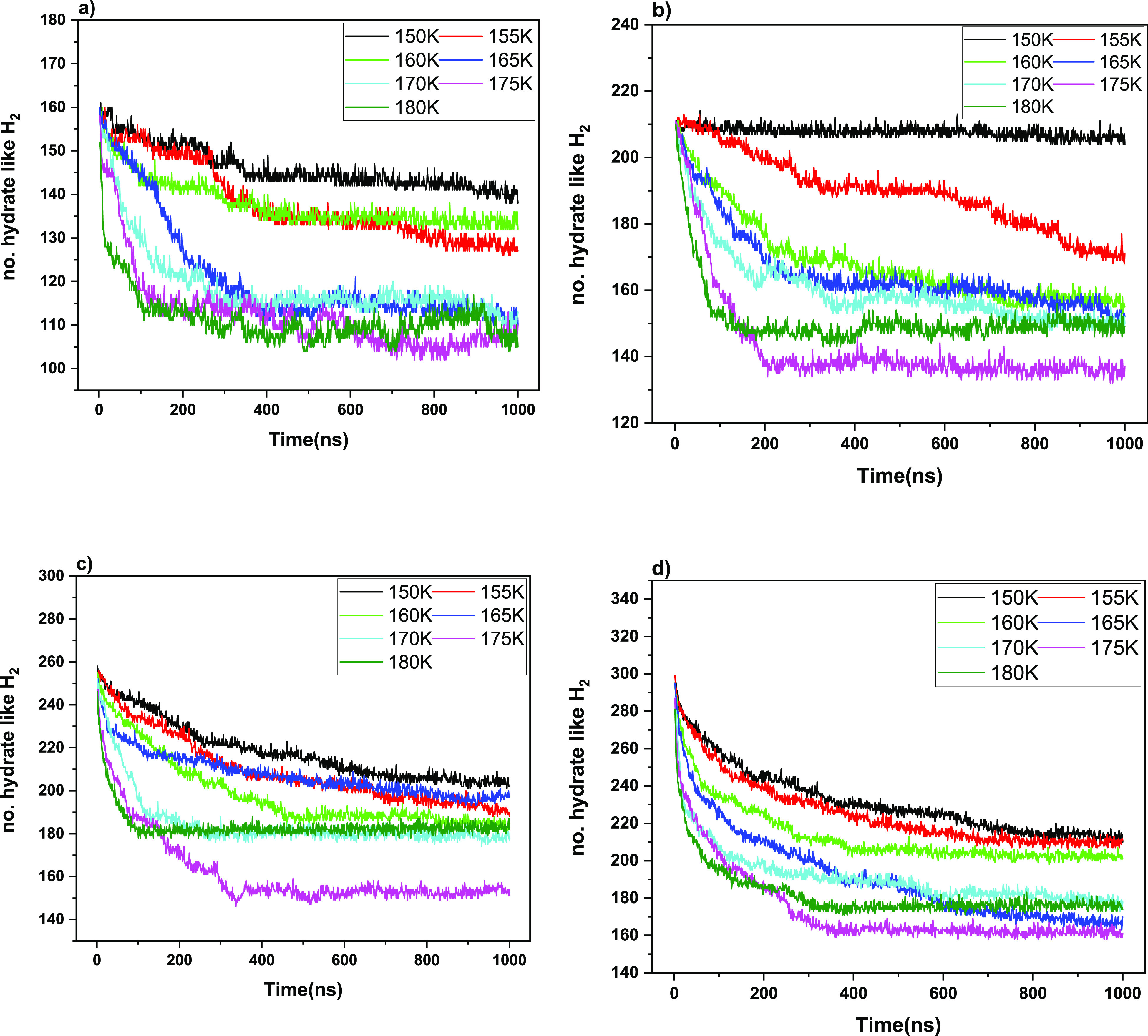
BC-classified hydratelike H_2_ count versus time, showing declathration and partial lattice emptying toward plateaux close to guest-fugacity equilibrium across the hydrate and now-gas phase (i.e., no longer vacuum). The results of the different 5^12^6^4^-cage occupancies are shown for (a) 1, (b) 2, (c) 3, and (d) 4.

**Figure 4:**
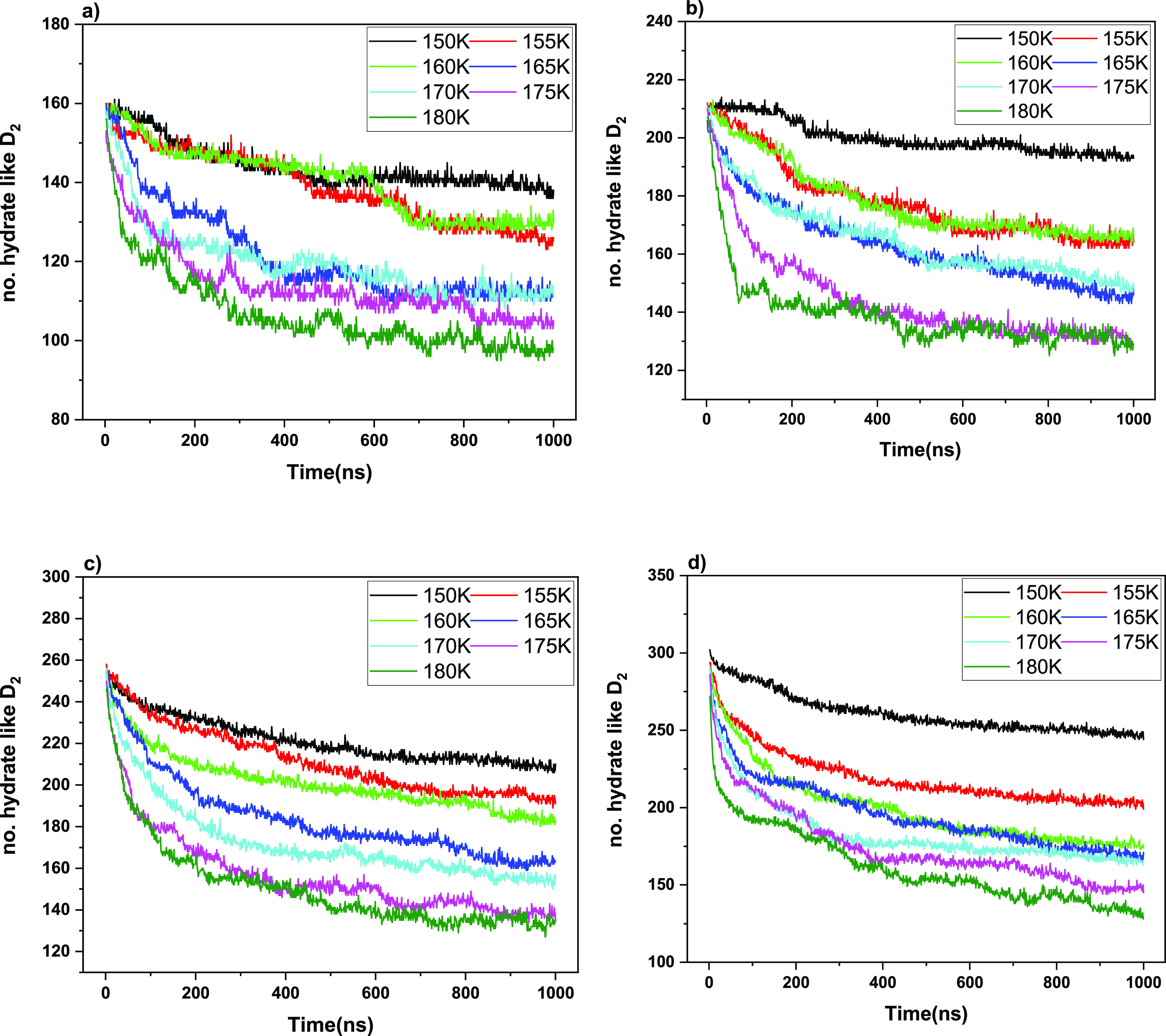
BC-classified hydratelike D_2_ count versus time, showing partial escape toward plateaux close to guest-fugacity equilibrium across the hydrate and now-gas phases (i.e., no longer vacuum). Different 5^12^6^4^-cage occupancies are shown: (a) 1, (b) 2, (c) 3, and (d) 4

The dynamics of guest release are shown in Figures 3 and 4 as a function of both initial nominal large-cage occupation and temperature, with the partial-escape leakage continuing until the H_2_/D_2_-fugacity difference between the hydrate and gas (initially vacuum) phases is very small. Of course, one may define the rate of interphase leakage/transfer in a manner consistent with Fick’s Law:
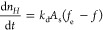
2where *H* refers to the guest (H_2_/D_2_), *A*_s_ is the cross-sectional surface area of a particle, *f*_e_ is the guest fugacity in the hydrate, and *f* is its level in the vacuum/developing-gas phase, with the guest fugacity (or, equivalently, chemical-potential) difference acting as the mass-transfer driving force.

It is clear for both H_2_ and D_2_ cases in Figures 3 and 4 that the decline of the driving force (cf. eq 2) as the leakage proceeds slows down the rate of mass transfer as one approaches the respective guest-concentration plateaux inside the hydrates, close to interphase thermodynamic equilibrium.

Although from Figures 3 and 4, it is clear that temperature affects the leakage rate, given that the escape from the hydrate lattice occurs by a cage-by-cage hopping mechanism, there is a less obviously dramatic effect of the initial (nominal) occupation. Certainly, in the case of quadruple large-cage occupation, the level of molecular “crowding” is quite elevated, given that there is a growing experimental and simulation body of consensus that large cages are typically no more than doubly occupied.^45^ Therefore, more elevated concentrations need to overcome larger free-energy barriers to jump from one cage to another, with all large cages having high occupation.^9,41,42,46,47^

The levels of the ultimate plateaux are different in each case in Figures 3 and 4, reflecting the different approximate fugacity balance of the guest in both hydrate and gas (initially vacuum) phases. On comparing Figure 3 (H_2_) versus Figure 4 (D_2_), although the rate of D_2_ leakage is generally somewhat slower than that of H_2_ (although understood in a first-order isotopic sense by double the mass), there is a greater ultimate reduction in D_2_ content vis-à-vis H_2_, ceteris paribus; this disparity becomes more marked at higher temperatures. As will be discussed below, the guest–cage interactions become more marked at higher temperatures, with a larger scope for greater-amplitude intracage rattling,^48^ together with faster intracage “tetrahedral-site-swapping” motions,^45^ and so heavier D_2_ molecules exhibit a greater degree of thermal activation, due to greater propensity for temperature-induced intracage vibrational and rattling activation. Although double in mass, the greater amplitude of D_2_ collisions with surrounding cages, and magnitude of momentum-transfer “leakage,” leads to cages’ greater “flexing” and time-dependent distortions, facilitating escape.

**Figure 5:**
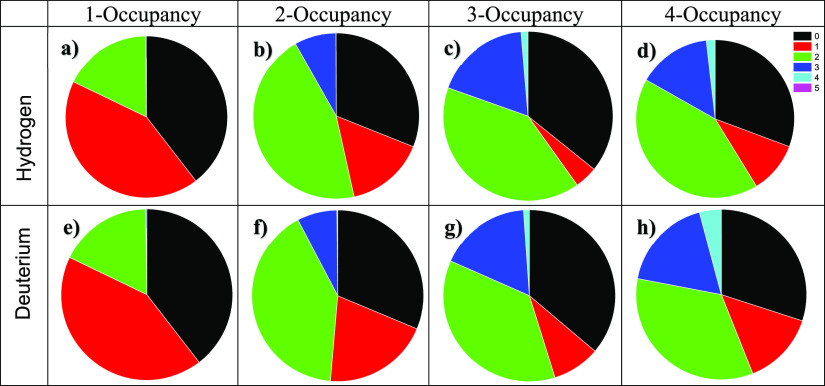
Large-cage occupancy overall percentage of the D_2_ and H_2_ in the latter part of simulations. (a-d) 1-, 2-, 3-, and 4-occpancy, respectively, for H_2_. (e-h) 1-, 2-, 3-, and 4-occupancy for D_2_.

Considering thermal effects to accelerate the kinetics of leakage, the activation energy (*E*_a_) for each system during this nonequilibrium leakage process was calculated for the non-steady-state rate constants (cf. eq 4) from Arrhenius-fitting Figures 3 and 4 at different temperatures (cf. Figures S3 and S4 and Tables S1 and S2, Supporting Information), and the values are summarized in Table 1. We can conclude that the nonequilibrium *E*_a_ within the clathrate hydrate is the lowest for the four-occupancy model, which is consistent with easier thermal activation of larger-cage occupancies.^48^ The highest *E*_a_ arises for double occupancy vis-à-vis other occupancies in both deuterium and hydrogen cases, and this is also supported by the occupancy percentage of the gas molecules. For nominal double occupation, refs (45). and (48) highlight the more highly stable intramolecular configurations in terms of the general tetrahedral structure vis-à-vis other occupancies (both thermodynamically and structurally and with respect to intra- and inter-cage-hopping propensity). This serves to rationalize the present findings for the highest activation energy during nonequilibrium molecular escape toward close to thermodynamic equilibrium, which was also reflected in longer cage dwell-time results for the nominal double-occupation case: this is clear in Figure 5, when considering probability distributions in terms of overall cage-residence times over the ensemble of guests. In large cages, nominally doubly occupied systems persist more in that double-occupation state in actuality—for H_2_, cf. Figure 5b vs 5a, c, and d and, for D_2_, cf. 5f vs 5e, g and h). This is also reflected in the allied tabulation in Table S3 (see the Supporting Information).

**Table 1:** Activation Energy of the H_2_ and D_2_ Leakage Rates (See Arrhenius Fits in Figures S3 and S4)

	hydrogen		deuterium	
name	*E*_a_ (kJ/mol)	Std error	*E*_a_ (kJ/mol)	Std error
1-occ	5.29	1.07	4.73	0.006
2-occ	8.08	2.67	6.98	1.31
3-occ	4.27	0.30	5.05	0.43
4-occ	3.85	0.26	3.59	0.48

**Figure 6:**
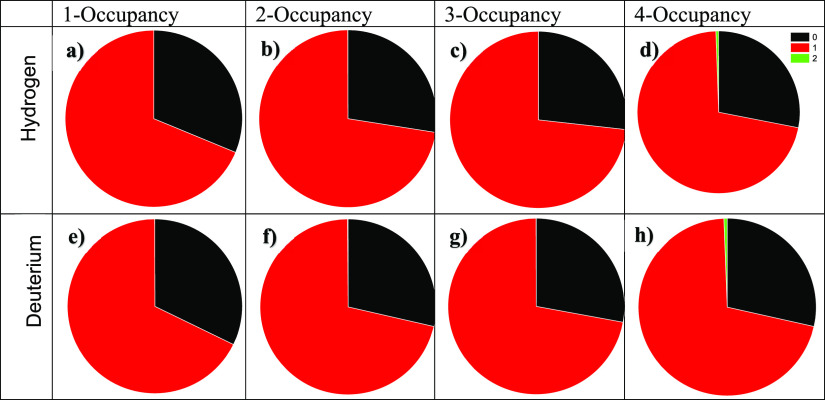
Small-cage occupancy overall percentage of the D_2_ and H_2_ in the latter part of simulations. (a-d) 1-, 2-, 3-, and 4-occpancy, respectively, for H_2_. (e-h) 1-, 2-, 3-, and 4-occupancy for D_2_.

**Figure 7:**
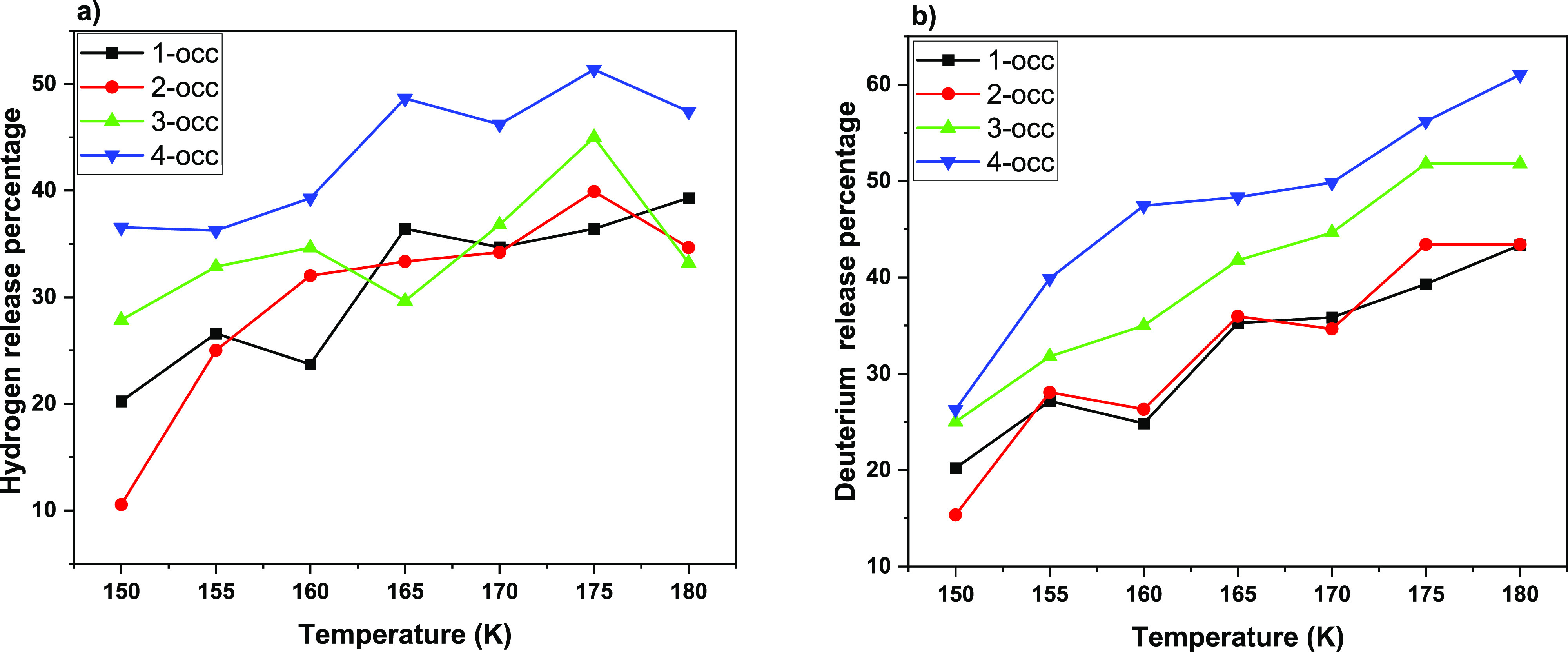
Release percentage versus temperature for all occupancies: (a) hydrogen and (b) deuterium.

Single occupancy is favored in small cages, and the results are shown in Figure 6 and in Table S2 (see the Supporting Information). The release percentages of H_2_ and D_2_ gases were calculated, and the release percentage increases with the increasing temperature and is shown in Figure 7. For the associated cage-occupation values, please see Tables S3 and S4 for hydrogen and deuterium, respectively. Figure 7 details the percentage release of the total amount of H_2_ and D_2_ in the systems as a function of temperature, which is broadly reflective of closer-to-equilibrium final plateaux encountered in the final release-dynamics plots of Figures 3 and 4 and is informed by the cage-occupancy details of Figures 5 and 6. In essence, and perhaps unsurprisingly, Figure 7 reveals that the quadruply occupied systems shed a greater proportion of their guests into the gas phase, given that the fugacity of the guests would be expected to be higher (and close to equal) in both the hydrate and gas phases for a larger number of guests. The most intriguing points of Figures 6 and 7 are the emptying of a greater proportion of the small cavities evident in the singly occupied 5^12^6^4^ case (which is also evident in Figure 6) and small cavities’ rare double occupation for initial quadruple occupation of large cages. Obviously, these distributions are sampled over a full microsecond of emptying, so Figures 5 and 6, in terms of time of occupancy-number distributions, must be noted as such.

Migration occurs in the main through hexagonal faces, as refs (45−48). make clear from free-energy-barrier considerations. However, rarely, it does occur through pentagonal faces (cf. Figure 6d,h), mostly for 5^12^ cages in the case of the (nominally) quadruply occupied large cages. The mechanism is essentially the same as through hexagonal cages, albeit, there is a much larger chemical-potential driving force in the case of quadruply occupied large cages, mostly to transfer through hexagonal faces to other larger cages and onward to the bulk vacuum/gas phase, but, in some cases, through pentagonal faces to a limited number of small 5^12^ cages.

**Figure 8:**
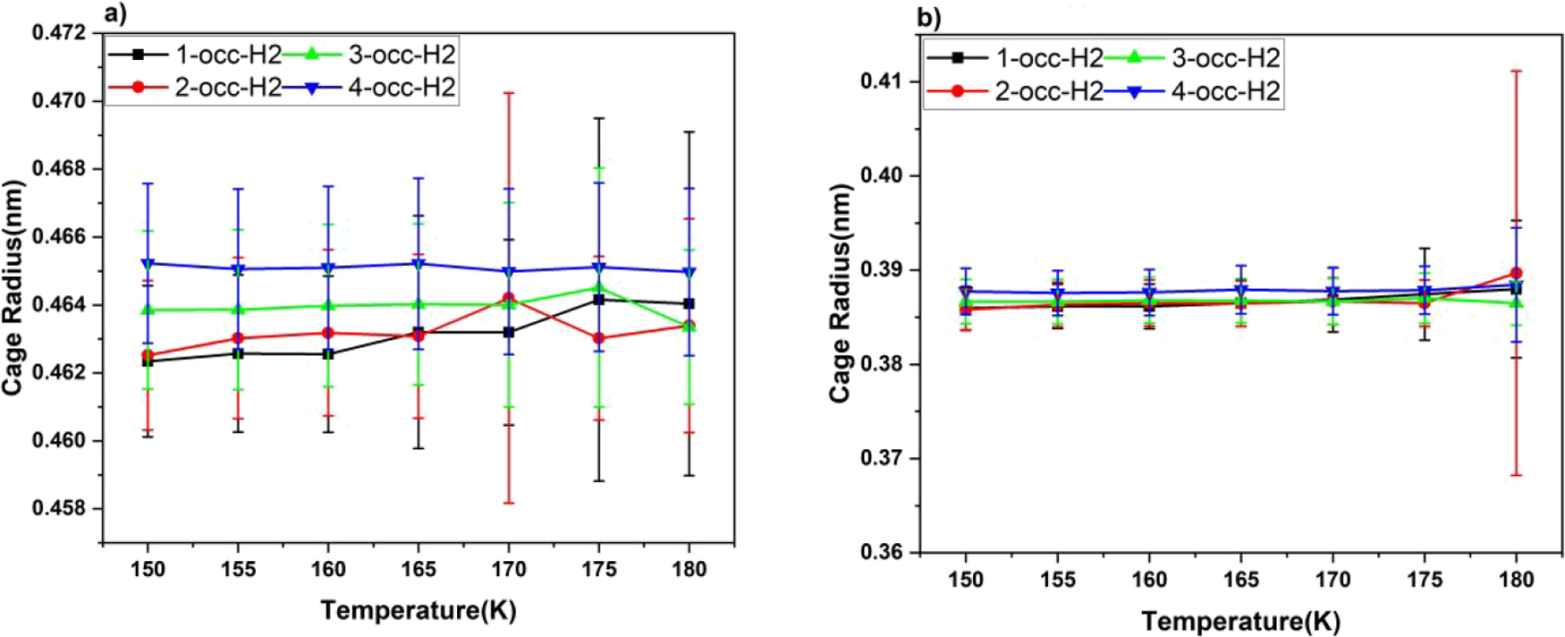
Average cage radius with respect to temperature for hydrogen (a) large cage and (b) small cage.

**Figure 9:**
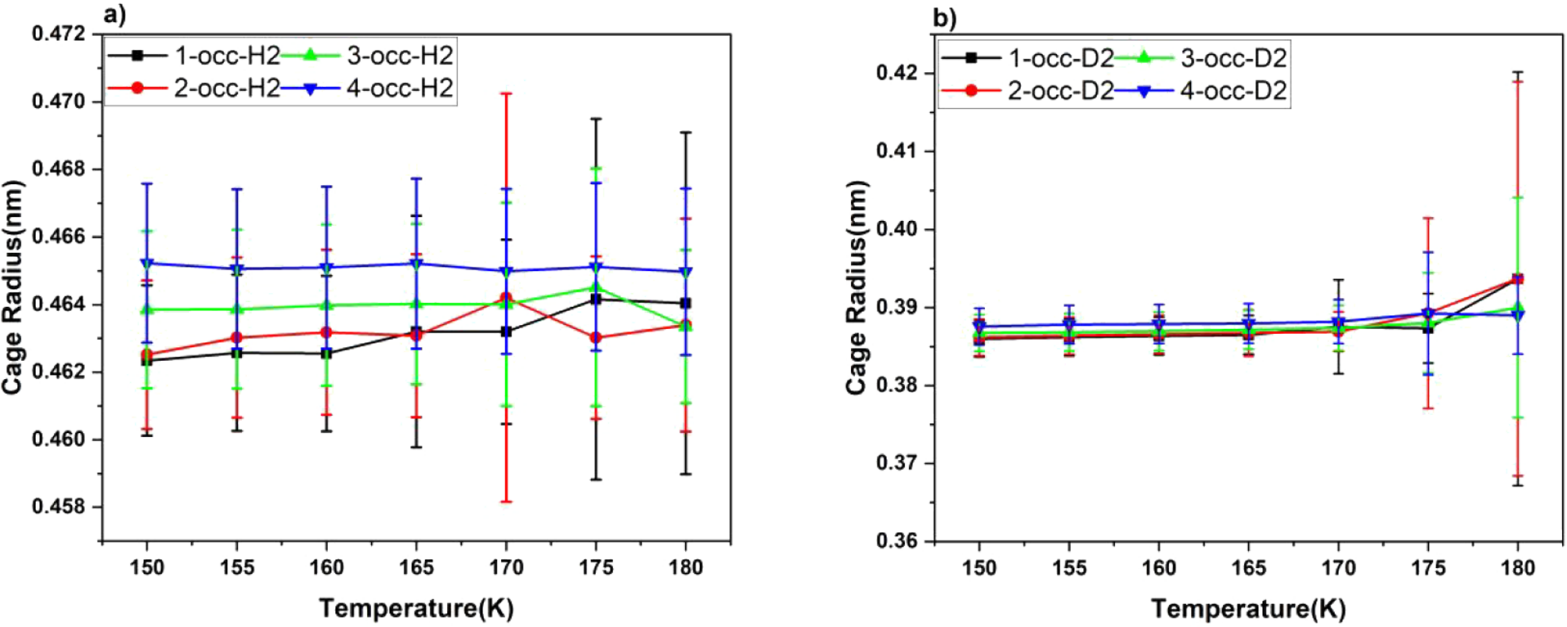
Averaged cage radius with respect to temperature for deuterium (a) large cage and (b) small cage.

Cage radii, both large and small, have been measured over the simulation period at different temperatures—see Figure 8 for H_2_ and Figure 9 for D_2_. As mentioned above in connection with the only somewhat slightly slower rate of D_2_-release kinetics (cf. Figure 3 vs Figure 4), this is rationalized by guest–cage interactions becoming more marked in their amplitude at higher temperatures. The cage radii in Figures 8 and 9, and more specifically their standard deviations, reveal vividly the greater-amplitude intracage rattling,^48^ together with faster intracage “tetrahedral-site-swapping” motions,^45^ and so the heavier D_2_ molecules exhibit greater collisions, facilitating their escape.

**Figure 10:**
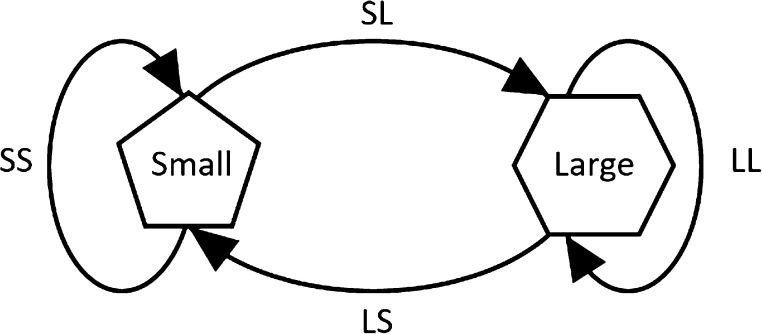
Schematic of a Markov model for internal cage hopping in the hydrate, adopted from ref (9).

Moving on to model the nonequilibrium emptying process in terms of a Markov process is instructive and illuminating in the case of the present results. A Markov chain is a series of trials in which only the immediate predecessor depends on the outcome of successive trials. A new state will be admitted in the Markov chain only if it is more favorable than the current state.^44^ This typically means that the new trial state is lower in energy in the sense of a simulation using an ensemble. If a guest molecule travels from a single cage to a secondary cage, one “trip” counts. Each such trip has been labeled based on the starting and the secondary cage type, as large to large (LL), large to small (LS), small to large (SL), and small to small (SS); a schematic is shown in Figure 10.^9^ From the Markov model, the hydrogen travel of the large-to-large (LL) cage is more favorable at all temperatures, despite the larger-amplitude thermal activation for D2 at thigh temperature (cf. cage radii in Figures 8 and 9—specifically high-temperature radius standard deviations). Most interestingly, for the deuterium, the large-to-large-cage travel is “encouraged” at low temperatures (with lesser thermal activation in D_2_-cage collisions, cf. Figure 8 vs Figure 9 and small-to-small is favorable when it reaches the highest temperature of the simulation, that is, 180 K—owing to the large level of distortions evident in the smaller cages at 180 K with more substantial D_2_-cage collisions (cf. large error bar on the bottom right of Figure 9). The tables of all occurrences are shown in Tables S5 and S6 (see the Supporting Information). As an example, Figure S5 illustrates the diffusion path of one H_2_ molecule through its journey leaving the hydrate structure, doing large-to-large cage hopping.

As we have shown in ref (48). with intercage energy-barrier estimates for H_2_ and D_2_ hopping, the lowering of temperature, alongside associated amplitudes of thermal vibrations of the guest and water cage-face molecules, facilitates especially large-to-large cage hopping transfer. This is based largely on geometric, steric, and negotiable-pathway considerations for these large-to-large cage transitions.

In terms of specific subpicosecond cage-centric dynamical phenomena, e.g., cage-rattling and distortion, these do play an important role, of course in cage-to-cage hopping within the hydrate phase.^41,42,45,48^ For instance, ref (41). considers cage-radii, distortion, flexing, and vibrational frequency for these processes before and during cage-hop events, while ref (48). considers guest intracage rattling and vibrational coupling with the water cage. Refs (42). and (45) consider free-energy barriers and thermodynamic aspects to cage-hopping, considering also important temperature and cage-occupation influences.

To investigate some thermodynamic aspects of lattice emptying, in and of itself an inherently nonequilibrium process, high-temperature binding free energies of guests were computed using the TI method, as outlined earlier, and sampled and averaged over molecular-hop events for the particular molecular guests in those respective individual cages (whether occupied singly or multiply). Given that the vast majority of the cage hopping took place in, and between, large cages, we considered these in our calculations for the different levels of occupation. However, given the observation of some small-to-small-cage hopping at higher temperatures, albeit more so for D_2_, we also examined such small-cage phenomena for single and quadruple 5^12^6^4^-cage occupations. Unsurprisingly, we have found that the guest–cage “binding” free energy (see Table 2) is larger for more crowded D_2_ in large cages, owing to greater-amplitude D_2_-cage collisions, with this also evident in small cages (especially for quadruple 5^12^6^4^-cage occupation, with the occasional “foray” into small cages for rare—but not-unprecedented—double occupation of small cages—cf. Figure 6h). Taken together, these free-energy observations are consistent with the thermodynamic ease or difficultly with which certain guests can transfer between different cage types—all given by the bigger “backdrop” of Fick’s Law driving toward equalizing as much as possible the guests’ fugacity, or chemical potential, in the gas and hydrate phases.

**Table 2:** Guest-Interaction Binding Free Energies in Large Cages and Selected Small Cages

		hydrogen		deuterium	
S. No.	name	small cage Δ*G* (KJ/mol)	large cage Δ*G* (KJ/mol)	small cage Δ*G* (KJ/mol)	large cage Δ*G* (KJ/mol)
1	1-occupancy	0.89	1.74	0.86	2.08
2	2-occupancy		10.11		8.52
3	3-occupancy		27.25		26.64
4	4-occupancy	4.63	54.64	3.38	45.22

## Conclusions

The leakage of both hydrogen (H_2_) and deuterium (D_2_) from sII clathrate hydrates, borne of guest chemical-potential equalization driving enhanced nonequilibrium intercage hopping, has been predicted by microsecond MD within the temperature range of 150–180 K—for which the hydrate lattice was found to be stable. In this pursuit, we considered initial large-cage (5^12^6^4^) guest occupancies of 1–4, with single occupation of 5^12^ cavities. Examining transient, nonequilibrium intercage hopping, we presented a diffusional activation energy for the four nominal large-cage occupancies (1–4) using leakage-rate fits. The intercage hopping of H_2_ and D_2_ was studied using Markov-chain models and expressed at different temperatures and large-cage occupancies. The free energy of guest “binding” in the large and small cages was also computed for all of the occupancies. Toward equilibrium, following the majority of H_2_/D_2_ escape via leakage, the percentage of occupancies was calculated for both H_2_ and D_2_ for all of the systems for all initial nominal occupancies; here, not unexpectedly, double occupancies occurred more favorably in large cages and single occupancies dominated in small cages.

We note that the prevalence of single- and, often, double-occupation of large cages, together with primarily single occupation of the small cages (cf. Figures 5 and 6) in the latter part of the simulations, close to the attainment of equilibrium. This suggests that the typical large-cage occupancies are of the order of 1.5–1.6 and 0.8–0.9 for small cages (subject, of course, to overall pressure and temperature levels). The calculated pressure values for the simulation boxes are reported in Table S7. This is in close accord with a body of more recent and growing theoretical and experimental cage-occupancy findings, as is discussed in refs (14). and (45). Indeed, with respect to the kinetic stability of cage occupation (i.e., kinetic suppression of leakage rate), in Figure 3b and Figure 4b for the respective doubly occupied H_2_ and D_2_ in 5^12^6^4^ cages, we note most tellingly that at 150 K, the number of enclathrated guests declines most slowly, especially for H_2_ (Figure 3b), emphasizing that double occupation also has kinetic aspects toward its relative stabilization, in agreement with the emerging consensus of experimental and theoretical literature studies, as discussed in refs (14). and (45).

In general, the D_2_ leakage rates were observed to be greater overall than those for H_2_. It is remarked that this is likely due to the stronger collisions between guest and cage molecules in the case of D_2_, which increases the likelihood of cage distortion and subsequent escape of the guests. As a suggestion for future study, a detailed study on the use of a flexible guest model with a nonrigid H–O–H or D–O–D bonds and its influence when considering the proportion of thermal activity can reveal more information about cage–guest interactions.

Given that hydrate–guest leakage is an inherently nonequilibrium diffusional process, MD simulation of both the present work and, indeed, for the case of neon declathration in ref (34)., needs to be allied further with experimental reports of the same guest-escape process. In this respect, in ref (34)., MD analyzed experimental measurements of ref (49). closely. Therefore, we both appeal to, and, indeed, challenge, the experimental community to investigate H_2_ and/or D_2_ release from either mixed or pure hydrates—based on our startling predictions herewith. Certainly, future theoretical work in this vein would do well to consider the currently MD-predicted H_2_/D_2_ leakage within the broader Pozhar–Gubbins statistical-mechanical theory of mass transport in strongly inhomogeneous fluids,^50,51^ as well as the framework of potential non-Onsager fluctuation-dissipation in mass-transfer flux,^52^ ideally in tandem with accurate guest-leakage experiments.

## Supporting Information Available

Snapshots of deuterium in clathrate hydrates, temperature conservations of trajectories, Arrhenius fits for the release and distance between guest molecules, and graphs; release rate percentages and cage occupancy percentage of deuterium and hydrogen molecules; and Markov-chain model and pressure values (PDF)

## References

[ref1] Sloan E. D. Jr.; Koh C. A.; Koh C. A. Clathrate Hydrates of Natural Gases; CRC Press, 2007.

[ref2] Chattaraj P. K.; Bandaru S.; Mondal S. Hydrogen Storage in Clathrate Hydrates. J. Phys. Chem. A 2011, 115, 187–193. 10.1021/jp109515a. 21155603

[ref3] English N. J.; El-Hendawy M. M.; Mooney D. A.; MacElroy J. M. D. Perspectives on Atmospheric CO2 Fixation in Inorganic and Biomimetic Structures. Coord. Chem. Rev. 2014, 269, 85–95. 10.1016/j.ccr.2014.02.015.

[ref4] Ahn Y.-H.; Moon S.; Koh D.-Y.; Hong S.; Lee H.; Lee J. W.; Park Y. One-Step Formation of Hydrogen Clusters in Clathrate Hydrates Stabilized via Natural Gas Blending. Energy Storage Mater. 2020, 24, 655–661. 10.1016/j.ensm.2019.06.007.

[ref5] Lauvergnat D.; Felker P.; Scribano Y.; Benoit D. M.; Bačić Z. H 2, HD, and D 2 in the Small Cage of Structure II Clathrate Hydrate: Vibrational Frequency Shifts from Fully Coupled Quantum Six-Dimensional Calculations of the Vibration-Translation-Rotation Eigenstates. J. Chem. Phys 2019, 150, 154303 10.1063/1.5090573. 31005099

[ref6] Dyadin Y. A.; Larionov E. G.; Manakov A. Y.; Zhurko F. V.; Aladko E. Y.; Mikina T. V.; Komarov V. Y. Clathrate Hydrates of Hydrogen and Neon. Mendeleev Commun. 1999, 9, 209–210.

[ref7] Mao W. L.; Mao H.; Goncharov A. F.; Struzhkin V. V.; Guo Q.; Hu J.; Shu J.; Hemley R. J.; Somayazulu M.; Zhao Y. Hydrogen Clusters in Clathrate Hydrate. Science (80-) 2002, 297, 2247–2249. 10.1126/science.1075394. 12351785

[ref8] Lokshin K. A.; Zhao Y.; He D.; Mao W. L.; Mao H. K.; Hemley R. J.; Lobanov M. V.; Greenblatt M. Structure and Dynamics of Hydrogen Molecules in the Novel Clathrate Hydrate by High Pressure Neutron Diffraction. Phys. Rev. Lett. 2004, 93, 1–4. 10.1103/PhysRevLett.93.125503. 15447276

[ref9] Krishnan Y.; Ghaani M. R.; Desmedt A.; English N. J. Hydrogen Inter-Cage Hopping and Cage Occupancies inside Hydrogen Hydrate: Molecular-Dynamics Analysis. Appl. Sci. 2021, 11, 282 10.3390/app11010282.

[ref10] Sebastianelli F.; Xu M.; Bačić Z. Quantum Dynamics of Small H2 and D2 Clusters in the Large Cage of Structure II Clathrate Hydrate: Energetics, Occupancy, and Vibrationally Averaged Cluster Structures. J. Chem. Phys. 2008, 129, 244706 10.1063/1.3049781. 19123525

[ref11] Xu M.; Sebastianelli F.; Bačić Z. Quantum Dynamics of H2, D2, and HD in the Small Dodecahedral Cage of Clathrate Hydrate: Evaluating H2 -Water Nanocage Interaction Potentials by Comparison of Theory with Inelastic Neutron Scattering Experiments. J. Chem. Phys. 2008, 128, 244715 10.1063/1.2945895. 18601373

[ref12] Tsimpanogiannis I. N.; Costandy J.; Kastanidis P.; El Meragawi S.; Michalis V. K.; Papadimitriou N. I.; Karozis S. N.; Diamantonis N. I.; Moultos O. A.; Romanos G. E.; et al. Economou, I. G. Using Clathrate Hydrates for Gas Storage and Gas-Mixture Separations: Experimental and Computational Studies at Multiple Length Scales. Mol Phys. 2018, 116, 2041–2060. 10.1080/00268976.2018.1471224.

[ref13] English N. J.; Tse J. S.; Carey D. Mechanisms for thermal conduction in various polymorphs of methane hydrate. Phys. Rev. B 2009, 80, 134306 10.1103/PhysRevB.80.134306. 19659158

[ref14] Ranieri U.; Koza M. M.; Kuhs W. F.; Gaal R.; Klotz S.; Falenty A.; Wallacher D.; Ollivier J.; Gillet P.; Bove L. E. Quantum Dynamics of H 2 and D 2 Confined in Hydrate Structures as a Function of Pressure and Temperature. J. Phys. Chem. C 2019, 123, 1888–1903. 10.1021/acs.jpcc.8b11606.

[ref15] Alavi S.; Klug D. D.; Ripmeester J. A. Simulations of Structure II H2 and D2 Clathrates: Potentials Incorporating Quantum Corrections. J. Chem. Phys 2008, 128, 064506 10.1063/1.2825618. 18282055

[ref16] Ma R.; Zhong H.; Liu J.; Zhong J.; Yan Y.; Zhang J.; Xu J. Molecular Insights into Cage Occupancy of Hydrogen Hydrate: A Computational Study. Processes 2019, 7, 1–12. 10.3390/pr7100699.

[ref17] Tsimpanogiannis I. N.; Economou I. G.; Stubos A. K. A Practical Methodology to Estimate the H2 Storage Capacity of Pure and Binary Hydrates Based on Monte Carlo Simulations. J. Chem. Eng. Data 2020, 65, 1289–1299. 10.1021/acs.jced.9b00707.

[ref18] Powers A.; Scribano Y.; Lauvergnat D.; Mebe E.; Benoit D. M.; Bačić Z. The Effect of the Condensed-Phase Environment on the Vibrational Frequency Shift of a Hydrogen Molecule inside Clathrate Hydrates. J. Chem. Phys 2018, 148, 144304 10.1063/1.5024884. 29655345

[ref19] Luis D. P.; Romero-Ramirez I. E.; González-Calderón A.; López-Lemus J. The Coexistence Temperature of Hydrogen Clathrates: A Molecular Dynamics Study. J. Chem. Phys. 2018, 148, 114503 10.1063/1.5017854. 29566510

[ref20] Russina M.; Kemner E.; Mezei F. Intra-Cage Dynamics of Molecular Hydrogen Confined in Cages of Two Different Dimensions of Clathrate Hydrates. Sci. Rep. 2016, 6, 1–8. 10.1038/2Fsrep27417. 27270444 PMC4895235

[ref21] Zhang Z.; Kusalik P. G.; Guo G. J. Molecular Insight into the Growth of Hydrogen and Methane Binary Hydrates. J. Phys. Chem. C 2018, 122, 7771–7778. 10.1021/acs.jpcc.8b00842.

[ref22] Valdés Á.; Kroes G. J. Theoretical Investigation of Two H 2 Molecules inside the Cages of the Structure H Clathrate Hydrate. J. Phys. Chem. C 2012, 116, 21664–21672. 10.1021/jp305742e.

[ref23] Hassanpouryouzband A.; Joonaki E.; Vasheghani Farahani M.; Takeya S.; Ruppel C.; Yang J.; English N. J.; Schicks J. M.; Edlmann K.; Mehrabian H.; et al. Gas Hydrates in Sustainable Chemistry. Chem. Soc. Rev. 2020, 49, 5225–5309. 10.1039/C8CS00989A. 32567615

[ref24] Alavi S.; Ripmeester J. A. Hydrogen-Gas Migration through Clathrate Hydrate Cages. Angew. Chemie - Int Ed. 2007, 46, 6102–6105. 10.1002/anie.200700250. 17623283

[ref25] Di Profio P.; Canale V.; Germani R.; Arca S.; Fontana A. Reverse Micelles Enhance the Formation of Clathrate Hydrates of Hydrogen. J. Colloid Interface Sci. 2018, 516, 224–231. 10.1016/j.jcis.2018.01.059. 29408108

[ref26] Härmas R.; Palm R.; Russina M.; Kurig H.; Grzimek V.; Härk E.; Koppel M.; Tallo I.; Paalo M.; Oll O.; et al. Transport Properties of H2 Confined in Carbide-Derived Carbons with Different Pore Shapes and Sizes. Carbon N. Y. 2019, 155, 122–128. 10.1016/j.carbon.2019.08.041.

[ref27] Strobel T. A.; Koh C. A.; Sloan E. D. Water Cavities of SH Clathrate Hydrate Stabilized by Molecular Hydrogen. J. Phys. Chem. B 2008, 112, 1885–1887. 10.1021/jp7110549. 18229920

[ref28] Wang Y.; Glazyrin K.; Roizen V.; Oganov A.; Chernyshov I.; Zhang X.; Greenberg E.; Prakapenka V. B.; Yang X.; Jiang S. Q.; Goncharov A. F. Novel Hydrogen Clathrate Hydrate. Phys. Rev. Lett 2020, 125, 255702. 33416341 10.1103/PhysRevLett.125.255702

[ref29] Strobel T.; Kim Y.; Koh C.; Sloan E. Clathrates of Hydrogen with Application towards Hydrogen Storage; ICGH, 2008.

[ref30] Russina M.; Guenther G.; Grzimek V.; Gainov R.; Schlegel M. C.; Drescher L.; Kaulich T.; Graf W.; Urban B.; Daske A.; et al. Upgrade Project NEAT′2016 at Helmholtz Zentrum Berlin – What Can Be Done on the Medium Power Neutron Source. Phys. B 2018, 551, 506–511. 10.1016/j.physb.2017.12.026.

[ref31] Strobel T. A.; Koh C. A.; Sloan E. D. Hydrogen Storage Properties of Clathrate Hydrate Materials. Fluid Phase Equilib. 2007, 261, 382–389. 10.1016/j.fluid.2007.07.028.

[ref32] Wang Y.; Yin K.; Fan S.; Lang X.; Yu C.; Wang S.; Li S. The Molecular Insight into the “Zeolite-Ice” as Hydrogen Storage Material. Energy 2021, 217, 119406 10.1016/j.energy.2020.119406.

[ref33] Yu C.; Fan S.; Lang X.; Wang Y.; Li G.; Wang S. Hydrogen and Chemical Energy Storage in Gas Hydrate at Mild Conditions. Int. J. Hydrogen Energy 2020, 45, 14915–14921. 10.1016/j.ijhydene.2020.03.228.

[ref34] Krishnan Y.; Ghaani M. R.; English N. J. Electric-Field Control of Neon Uptake and Release to and from Clathrate Hydrates. J. Phys. Chem. C 2019, 123, 27554–27560. 10.1021/acs.jpcc.9b07257.

[ref35] Pronk S.; Páll S.; Schulz R.; Larsson P.; Bjelkmar P.; Apostolov R.; Shirts M. R.; Smith J. C.; Kasson P. M.; van der Spoel D.; et al. GROMACS 4.5: A High-Throughput and Highly Parallel Open Source Molecular Simulation Toolkit. Bioinformatics 2013, 29, 845–854. 10.1093/bioinformatics/btt055. 23407358 PMC3605599

[ref36] Lindahl E.; Hess B.; van der Spoel D. GROMACS 3.0: A Package for Molecular Simulation and Trajectory Analysis. J. Mol. Model. 2001, 7, 306–317. 10.1007/s008940100045.

[ref37] Van Der Spoel D.; Lindahl E.; Hess B.; Groenhof G.; Mark A. E.; Berendsen H. J. C. GROMACS: Fast, Flexible, and Free. J. Comput. Chem. 2005, 26, 1701–1718. 10.1002/jcc.20291. 16211538

[ref38] Hess B.; Kutzner C.; van der Spoel D.; Lindahl E. GROMACS 4: Algorithms for Highly Efficient, Load-Balanced, and Scalable Molecular Simulation. J. Chem. Theory Comput. 2008, 4, 435–447. 10.1021/ct700301q. 26620784

[ref39] Alavi S.; Ripmeester J. A.; Klug D. D. Molecular-Dynamics Study of Structure II Hydrogen Clathrates. J. Chem. Phys. 2005, 123, 024507 10.1063/1.1953577. 16050759

[ref40] Ghesquière P.; Mineva T.; Talbi D.; Theulé P.; Noble J. A.; Chiavassa T. Diffusion of Molecules in the Bulk of a Low Density Amorphous Ice from Molecular Dynamics Simulations. Phys. Chem. Chem. Phys. 2015, 17, 11455–11468. 10.1039/C5CP00558B. 25854329

[ref41] Cao H.; English N. J.; MacElroy J. M. D. Diffusive Hydrogen Inter-Cage Migration in Hydrogen and Hydrogen-Tetrahydrofuran Clathrate Hydrates. J. Chem. Phys. 2013, 138, 094507 10.1063/1.4793468. 23485313

[ref42] Burnham C. J.; English N. J. Free-Energy Calculations of the Intercage Hopping Barriers of Hydrogen Molecules in Clathrate Hydrates. J. Phys. Chem. C 2016, 120, 16561–16567. 10.1021/acs.jpcc.6b06524.

[ref43] Lu Q.; He X.; Hu W.; Chen X.; Liu J. Stability, Vibrations, and Diffusion of Hydrogen Gas in Clathrate Hydrates: Insights from Ab Initio Calculations on Condensed-Phase Crystalline Structures. J. Phys. Chem. C 2019, 123, 12052–12061. 10.1021/acs.jpcc.8b11586.

[ref44] Ferdows M.; Ota M. Molecular Simulation Study for CO2 Clathrate Hydrate. Chem. Eng. Technol. 2005, 28, 168–173. 10.1002/ceat.200407056.

[ref45] English N. J.; Burnham C. J. Intra-Cage Structure, Vibrations and Tetrahedral-Site Hopping of H_2_ and D_2_ in Doubly-Occupied 5^12^6^4^ Cages in SII Clathrate Hydrates from Path-Integral and Classical Molecular Dynamics. Appl. Sci. 2021, 11, 1–9.

[ref46] Cendagorta J. R.; Powers A.; Hele T. J. H.; Marsalek O.; Bačić Z.; Tuckerman M. E. Competing Quantum Effects in the Free Energy Profiles and Diffusion Rates of Hydrogen and Deuterium Molecules through Clathrate Hydrates. Phys. Chem. Chem. Phys. 2016, 18, 32169–32177. 10.1039/C6CP05968F. 27849073

[ref47] Cendagorta J. R.; Shen H.; Bačić Z.; Tuckerman M. E. Enhanced Sampling Path Integral Methods Using Neural Network Potential Energy Surfaces with Application to Diffusion in Hydrogen Hydrates. Adv. Theory Simul. 2020, 2000258 10.1002/adts.202000258.

[ref48] Burnham C. J.; Futera Z.; English N. J. Quantum and Classical Inter-Cage Hopping of Hydrogen Molecules in Clathrate Hydrate: Temperature and Cage-Occupation Effects. Phys. Chem. Chem. Phys. 2017, 19, 717–728. 10.1039/C6CP06531G. 27921106

[ref49] Falenty A.; Hansen T. C.; Kuhs W. F. Formation and Properties of Ice XVI Obtained by Emptying a Type SII Clathrate Hydrate. Nature 2014, 516, 231–233. 10.1038/nature14014. 25503235

[ref50] Pozhar L. A.; Gubbins K. E. Transport Theory of Dense, Strongly Inhomogeneous Fluids. J. Chem. Phys. 1993, 99, 8970–8996. 10.1063/1.465567.

[ref51] Pozhar L. A.; Gubbins K. E. Quasihydrodynamics of Nanofluid Mixtures. Phys. Rev. E 1997, 56, 5367–5396. 10.1103/PhysRevE.56.5367.

[ref52] Sieniutycz S. Entropy of Flux Relaxation and Variational Theory of Simultaneous Energy and Mass Transport Governed by Non-Onsager Phenomenological Equations. Appl. Sci. Res. 1982, 39, 87–103. 10.1007/BF00457012.

