# Supplementary material for: Hydrogen and Deuterium Molecular Escape from Clathrate Hydrates: “Leaky” Microsecond-Molecular-Dynamics Predictions
Source: J Phys Chem C Nanomater Interfaces. 2021 Apr 9;125(15):8430–9. doi: 10.1021/acs.jpcc.1c00987 (PMC8279647; doi:10.1021/acs.jpcc.1c00987)
Supplement: Supplementary file 1 — jp1c00987_si_001.pdf [file jp1c00987_si_001.pdf]

Supporting Information

for

Hydrogen and Deuterium Molecular Escape from Clathrate Hydrates: 'Leaky' Microsecond-Molecular-Dynamics Predictions

Yogeshwaran Krishnan, Mohammad Reza Ghaani* and Niall J. English*

School of Chemical and Bioprocess Engineering, University College Dublin, Belfield, Dublin 4, Ireland

Correspondence: mohammad.ghaani@ucd.ie (M.R.G.); Tel.: +353-1-716-1758 (M.R.G.); niall.english@ucd.ie (N.J.E.); Tel.: +353-1-716-1646 (N.J.E.); Fax: +353-1-716-1177 (N.J.E.)

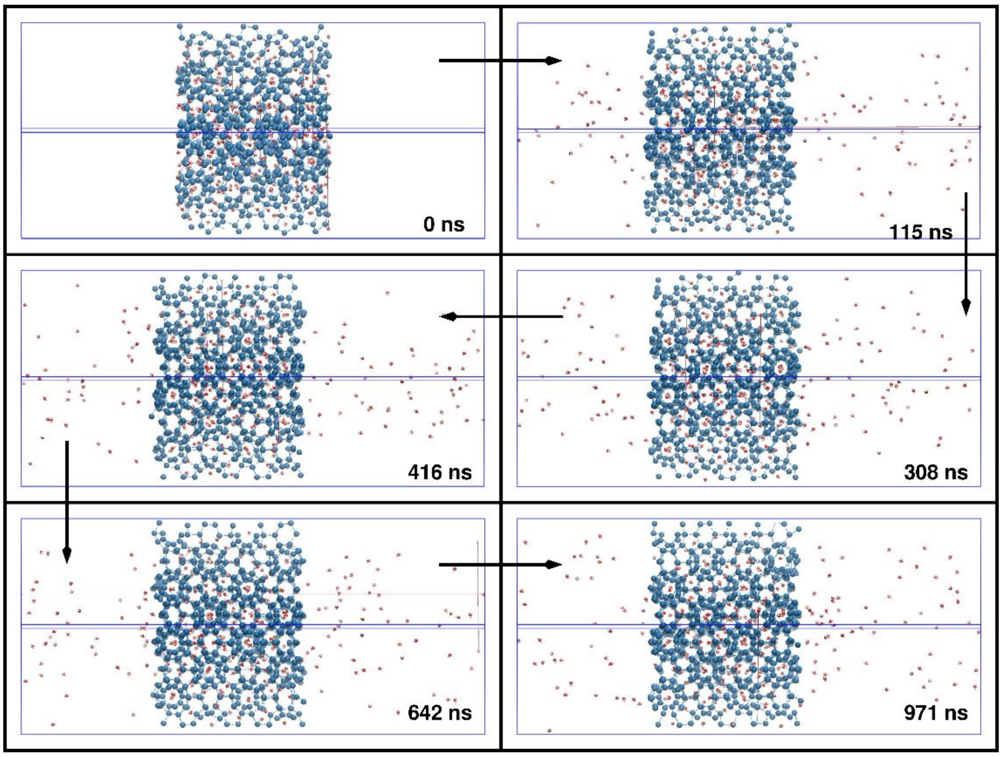

Figure S1. Snapshots of D_2 release in from (doubly occupied $5^{12}6^4$-cage) clathrate hydrate. Red denotes hydrogen, with release into the vacuum. Blue represents BC-classified hydrate-like water molecules in the hydrate phase.

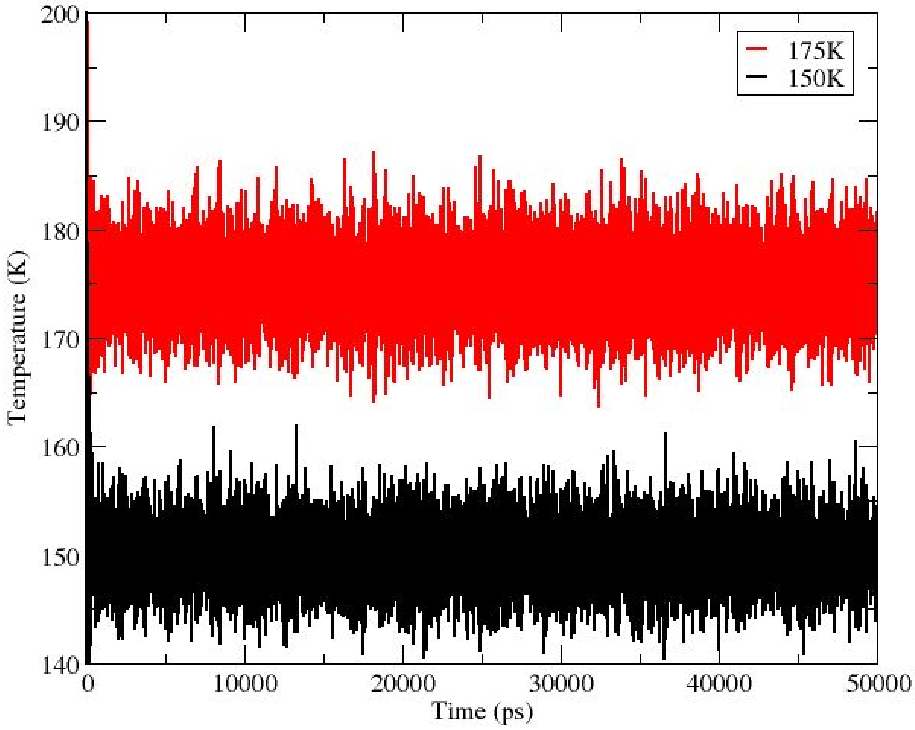

Figure S2. Temperature versus time for (doubly-occupied $5^{12}6^4$-cage) clathrate hydrate.

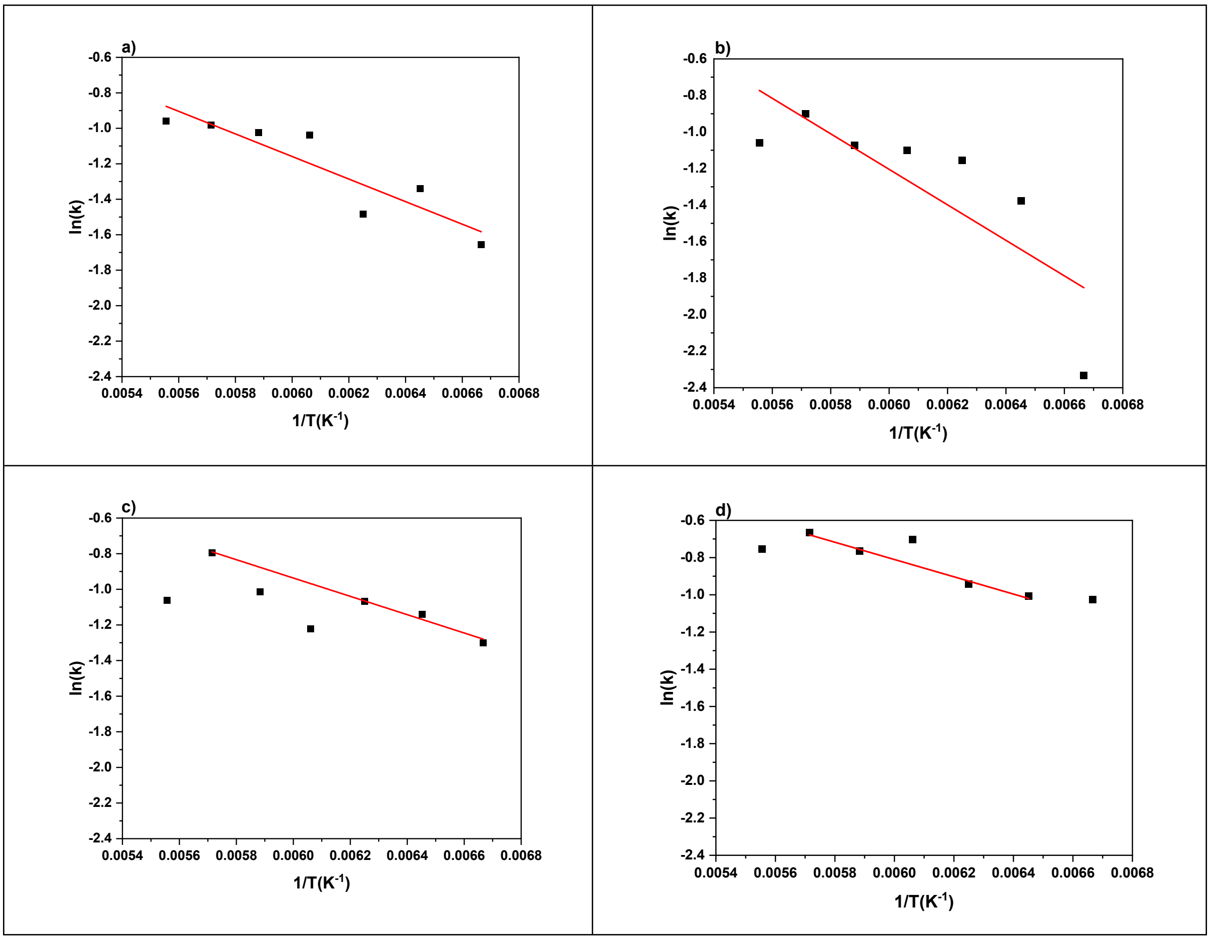

Figure S3. Activation-energy Arrhenius fits for the release rate fittings of hydrogen molecules (cf. Fig. 3 and Table S1) for (a) 1-occupancy, (b) 2-occupancy, (c) 3-occupancy and (d) 4-occupancy

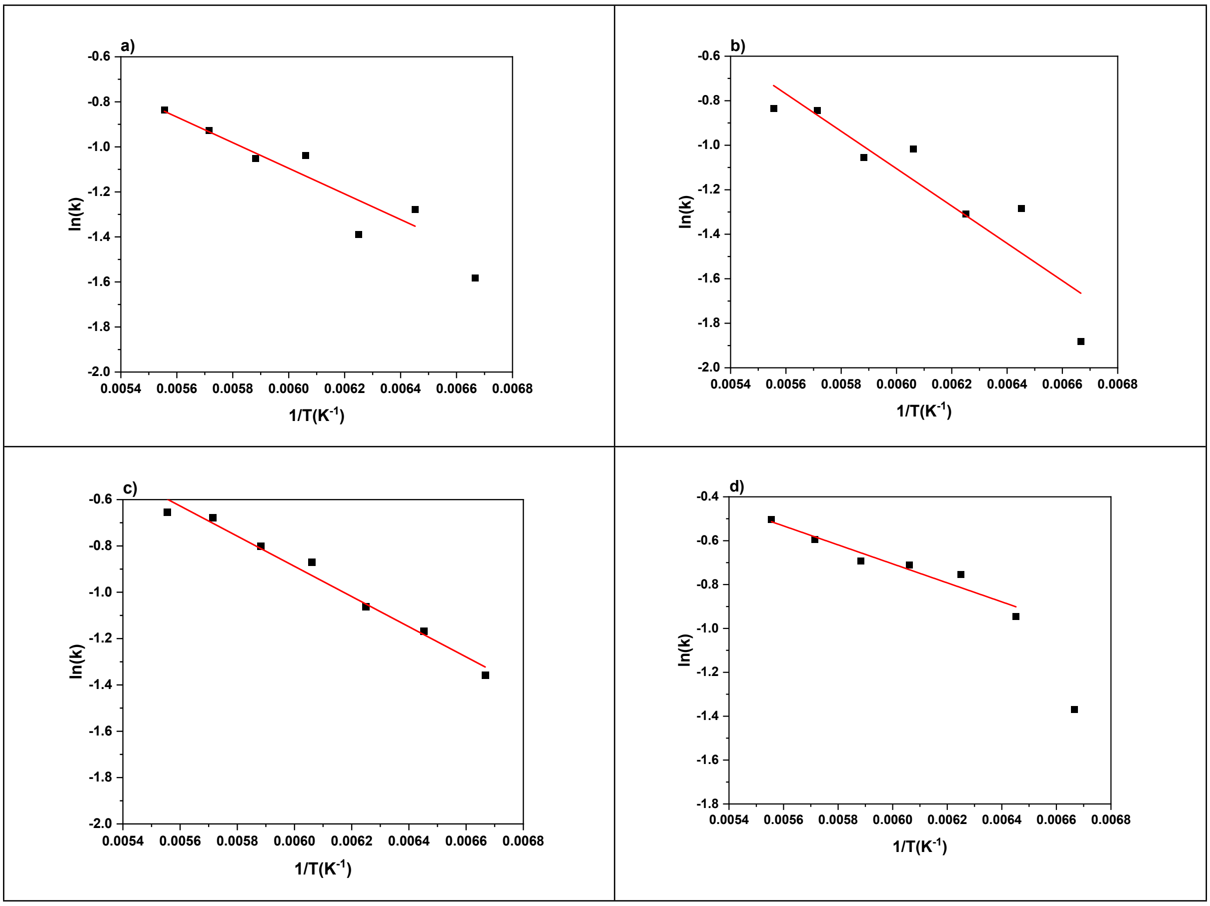

Figure S4. Activation-energy Arrhenius fits for the release rate fittings of D_2 molecules (cf. Fig. 4 and Table S2) for (a) 1-occupancy, (b) 2-occupancy, (c) 3-occupancy and (d) 4-occupancy

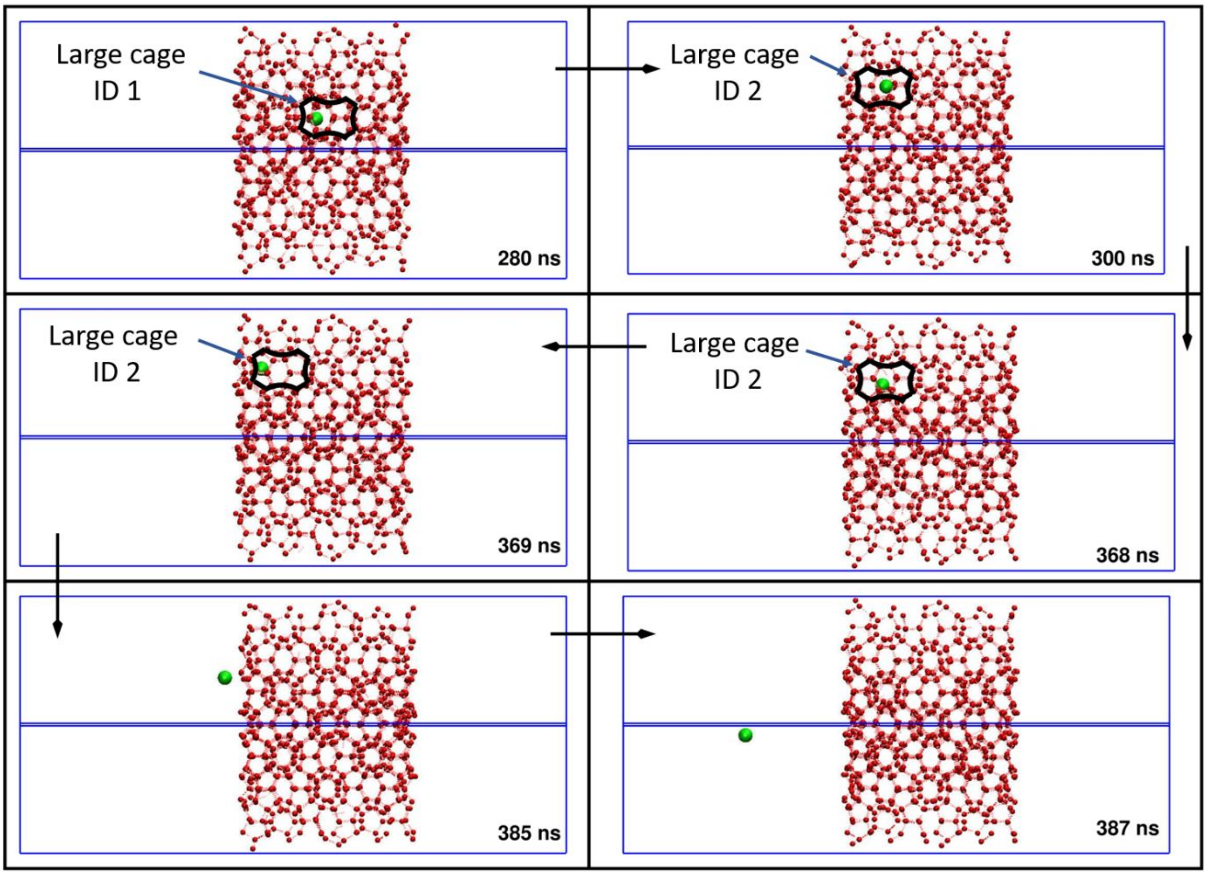

Figure S5. A representative example of a diffusion path of one encapsulated hydrogen molecule. The molecule belonged to a large cage (cage ID 1), and, after about 20 ns residence there, it jumped ('large-to-large') to a neighbouring large cage (cage ID 2), staying there for around 80 ns, and leave the hydrate structure after that.

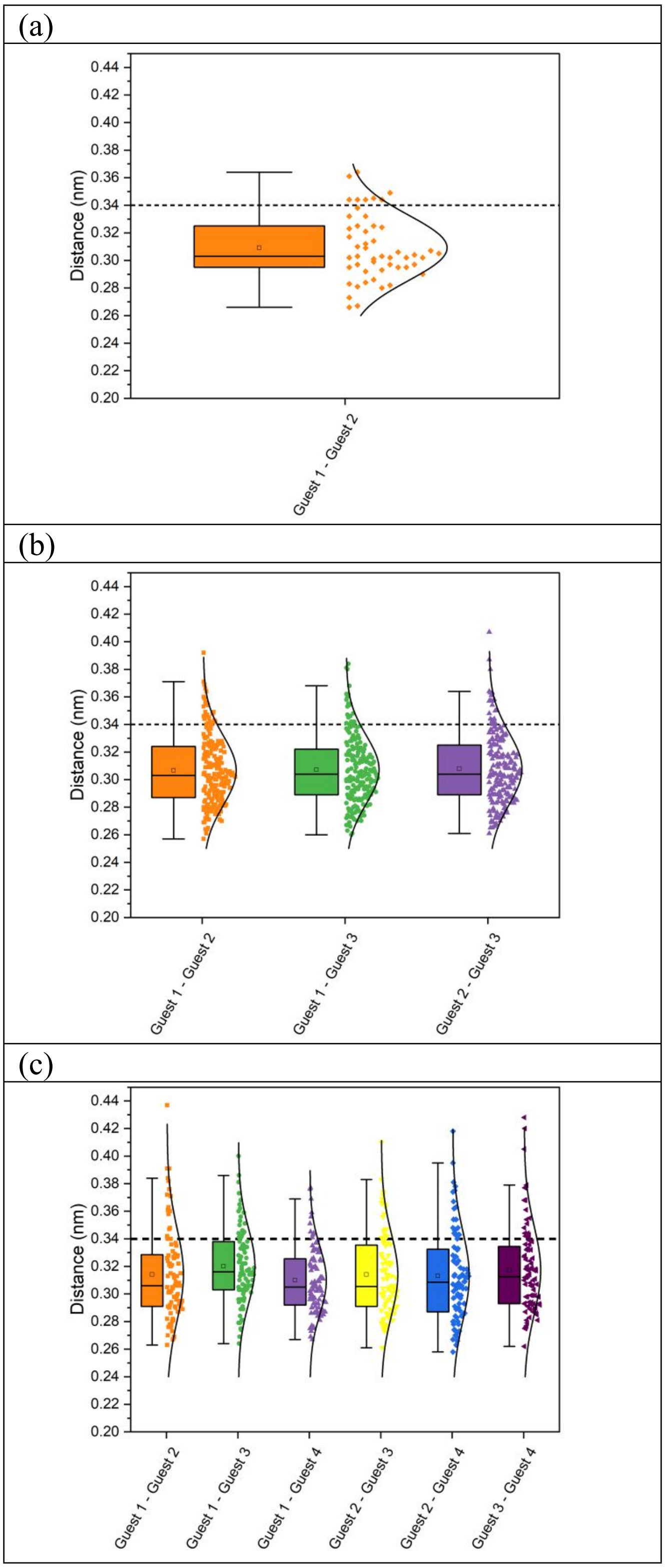

Figure S6. Pair-distance distribution sampled over 100 ns between the guest molecules in one large cage in case of 2-occ (a), 3-occ (b) and 4-occ (c). The values below the horizontal dashed line, represent repulsive force between the molecules, while for distances larger than 0.34 nm, largely attractive forces are experienced by the molecules. Broad distribution over the observed distances during 100 ns simulation suggest relatively free movement of the guest molecules inside the large cages.

Table S1. Release rates of hydrogen molecule in clathrate hydrate (cf. eqn. 2, Fig. 3)

Temperature (K)	1-occ	2-occ	3-occ	4-occ
150	20.23	10.52	27.85	36.55
155	26.58	25.00	32.85	36.25
160	23.69	32.01	34.64	39.27
165	36.41	33.33	29.64	48.64
170	34.68	34.21	36.78	46.22
175	36.41	39.91	45.00	51.35
180	39.30	34.64	33.21	47.43

Table S2. Release rates of D_2 molecule in clathrate hydrate (cf. eqn. 2, Fig. 4)

Temperature (K)	1-occ	2-occ	3-occ	4-occ
150	20.23	15.35	25.00	26.28
155	27.16	28.07	31.78	39.87
160	24.85	26.31	35.00	47.43
165	35.26	35.96	41.78	48.33
170	35.83	34.64	44.64	49.84
175	39.30	43.42	51.78	56.19
180	43.35	43.42	51.78	61.02

Table S3. Cage occupancy overall percentage of the D_2 and H_2 in large cages

D_2 large cage occupancy in percentage (%)					H_2 large cage occupancy in percentage (%)			
Occupancies	1-occ	2-occ	3-occ	4-occ	1-occ	2-occ	3-occ	4-occ
0	39.53	31.31	36.18	30.00	39.53	31.06	35.80	30.71
1	42.61	20.04	9.01	13.96	42.61	15.47	4.35	10.55
2	17.63	40.96	36.40	34.02	17.63	45.29	40.25	41.91
3	0.20	7.52	17.29	17.84	0.20	8.11	18.24	14.99
4	0	0.14	1.09	4.13	0	0.06	1.33	1.79
5	0	0.0025	0.001	0.02	0	0	0	0.018

Table S4. Cage occupancy overall percentage of the D_2 and H_2 in small cages

D_2 small cage occupancy in percentage (%)					H_2 small cage occupancy in percentage (%)			
Occupancies	1-occ	2-occ	3-occ	4-occ	1-occ	2-occ	3-occ	4-occ
0	32.19	28.56	27.87	28.52	31.22	27.46	26.77	28.07
1	67.71	71.24	72.06	70.87	68.77	72.41	73.22	71.38
2	0.043	0.11	0.02	0.59	0.001	0.041	0.0001	0.541

Table S5. Markov-chain model for hydrogen hopping

1-occ-H_2	150K	155K	160K	165K	170K	175K	180K
LL	2	0	5	8	25	45	67
LS	0	0	0	0	4	4	18
SL	0	0	0	0	6	3	11
SS	0	0	0	0	3	16	15
2-occ							
LL	50	71	89	108	134	67	117
LS	0	0	0	0	0	0	1
SL	0	0	0	0	0	0	0
SS	0	0	0	0	0	0	632
3-occ							
LL	290	323	303	376	285	368	167
LS	0	0	0	0	0	0	0
SL	0	0	0	0	0	0	0
SS	0	0	0	0	0	0	0
4-occ							
LL	513	398	406	408	315	332	380
LS	0	1	0	0	0	1	1
SL	0	0	0	0	0	0	0
SS	4	3	1	6	0	3	3

Table S6. Markov-chain model for deuterium hopping

1-occ-D_2	150K	155K	160K	165K	170K	175K	180K
LL	0	3	4	8	11	5	20
LS	0	0	0	0	0	0	13
SL	0	0	0	0	0	0	28
SS	0	0	0	0	4	3	635
2-occ							
LL	66	89	76	85	166	179	230
LS	0	0	0	0	0	0	83
SL	0	0	0	0	0	0	158
SS	0	0	0	0	0	129	888
3-occ							
LL	257	285	283	278	279	229	322
LS	0	0	0	0	0	5	103
SL	0	0	0	0	0	7	203
SS	0	0	0	0	0	48	941
4-occ							
LL	327	437	426	438	348	435	354
LS	0	0	1	0	0	2	0
SL	0	0	0	0	0	5	0
SS	3	1	3	2	6	37	19

Table S7. Calculated pressure in the simulation box of different models with various cage occupancy

Temperature (K)	Pressure (bar)							
	1-Occupancy		2-Occupancy		3-Occupancy		4-Occupancy	
	D_2	H_2	D_2	H_2	D_2	H_2	D_2	H_2
150	-74.10	-65.06	-43.65	-64.31	-107.26	-114.82	-256.12	-227.11
155	-55.74	-43.21	-55.00	-57.70	-102.15	-114.09	-259.46	-260.11
160	-34.78	-39.19	-23.43	-43.43	-98.83	-70.86	-206.72	-208.09
165	-29.32	-26.93	-40.86	-26.55	-71.39	-78.09	-205.72	-214.24
170	-34.59	-32.75	-27.96	-30.59	-83.72	113.79	-207.88	-207.34
175	-14.74	-46.24	-13.99	-8.52	-57.03	-56.19	-184.59	-186.67
180	-27.61	-34.31	2.79	-11.84	-61.57	-55.06	-188.83	-206.68

Full references

List of complete authors for a few references that have more than 10 authors are listed below.

- Reference 12 (main text)
  Tsimpanogiannis, I. N.; Costandy, J.; Kastanidis, P.; El Meragawi, S.; Michalis, V. K.; Papadimitriou, N. I.; Karozis, S. N.; Diamantonis, N. I.; Moultos, O. A.; Romanos, G. E.; Stubos, A. K.; Economou, I. G. Using Clathrate Hydrates for Gas Storage and Gas-Mixture Separations: Experimental and Computational Studies at Multiple Length Scales. Mol. Phys. 2018, 116 (15–16), 2041–2060. https://doi.org/10.1080/00268976.2018.1471224.
- Reference 23 (main text)
  Hassanpouryouzband, A.; Joonaki, E.; Vasheghani Farahani, M.; Takeya, S.; Ruppel, C.; Yang, J.; English, N. J.; Schicks, J. M.; Edlmann, K.; Mehrabian, H.; Aman, Z. M.; Tohidi, B. Gas Hydrates in Sustainable Chemistry. Chem. Soc. Rev. 2020, 49 (15), 5225–5309. https://doi.org/10.1039/c8cs00989a.
- Reference 26 (main text)
  Härmas, R.; Palm, R.; Russina, M.; Kurig, H.; Grzimek, V.; Härk, E.; Koppel, M.; Tallo, I.; Paalo, M.; Oll, O.; Embs, J.; Lust, E. Transport Properties of H2 Confined in Carbide-Derived Carbons with Different Pore Shapes and Sizes. Carbon N. Y. 2019, 155, 122–128. https://doi.org/10.1016/j.carbon.2019.08.041.

- Reference 30 (main text)
  Russina, M.; Guenther, G.; Grzimek, V.; Gainov, R.; Schlegel, M. C.; Drescher, L.; Kaulich, T.; Graf, W.; Urban, B.; Daske, A.; Grotjahn, K.; Hellhammer, R.; Buchert, G.; Kutz, H.; Rossa, L.; Sauer, O. P.; Fromme, M.; Wallacher, D.; Kiefer, K.; Klemke, B.; Grimm, N.; Gerischer, S.; Tsapatsaris, N.; Rolfs, K. Upgrade Project NEAT'2016 at Helmholtz Zentrum Berlin – What Can Be Done on the Medium Power Neutron Source. Phys. B Condens. Matter 2018, 551 (October 2017), 506–511.
- https://doi.org/10.1016/j.physb.2017.12.026.

- Reference 35 (main text)
  Pronk, S.; Páll, S.; Schulz, R.; Larsson, P.; Bjelkmar, P.; Apostolov, R.; Shirts, M. R.; Smith, J. C.; Kasson, P. M.; van der Spoel, D.; Hess, B.; Lindahl, E. GROMACS 4.5: A High-Throughput and Highly Parallel Open-Source Molecular Simulation Toolkit. Bioinformatics 2013, 29 (7), 845–854. https://doi.org/10.1093/bioinformatics/btt055.
